# SCKM: Symmetric Co-Skew Moment for User Selection in Federated Learning

**DOI:** 10.3390/e28060630

**Published:** 2026-06-02

**Authors:** Liangyan Li, Yangyi Liu, Yimo Ning, Stefano Rini, Jun Chen

**Affiliations:** 1 Huawei Technologies Co., Ltd., Waterloo, ON N2J 4PP, Canada; 2 Department of Electrical and Computer Engineering, McMaster University, Hamilton, ON L8S 4L8, Canada; yangyiliu21@gmail.com (Y.L.); chenjun@mcmaster.ca (J.C.); 3 Department of Computer Science, University of Toronto, Toronto, ON M5S 1A1, Canada; yimo.ning@mail.utoronto.ca; 4 Department of Electronics and Electrical Engineering, National Yang Ming Chiao Tung University, Hsinchu 300, Taiwan; stefano.rini@nycu.edu.tw

**Keywords:** federated learning, client selection, non-IID data, informational redundancy, semantic compression, communication-efficient federated learning

## Abstract

We introduce the *symmetric co-skewness moment* (SCKM)—a third-order informational dissimilarity metric that consistently outperforms state-of-the-art client-selection heuristics in federated learning (FL) under heterogeneous data. Unlike similarity-driven schemes, SCKM minimizes redundancy by favoring clients with complementary gradients, delivering faster and more stable convergence even at high heterogeneity levels. Operating on highly compressed 0.5% gradient summaries, our framework provides two operating modes for different deployment scales: (i) SCKM-Select directly ranks and schedules a small candidate pool, whereas (ii) SCKM-Cluster adds a fast, elbow-guided clustering step to scalably choose from thousands of users. We evaluate both variants on a VGG-16 model across multiple non-IID partition schemes and initializations, observing consistent gains over leading cosine-similarity, loss-sketch, and max-diversity baselines—without increasing the communication budget.

## 1. Introduction

Federated learning (FL) [1] enables a population of distributed devices and edge nodes to collaboratively train a shared model without exporting raw data. A server simply aggregates the locally computed updates to refine the global model. In practice, however, FL contends with three intertwined hurdles: (i) statistical heterogeneity, arising from private, non-IID data distributions; (ii) communication limits, which restrict the fraction of clients that can upload updates per round; and (iii) participation imbalance, whereby rare-class clients are chronically under-selected, harming fairness and accuracy. Consequently, client data is typically non-IID, leading to unstable convergence and biased global models when using naive aggregation strategies like FedAvg [1]. To mitigate these issues, *client selection* has become essential for improving both communication efficiency and optimization quality. While early approaches focused primarily on system availability or computational constraints, recent methods prioritize statistical utility. Popular strategies select clients based on criteria such as data representativeness [2], update magnitudes [3], or local losses [4]. More sophisticated gradient-based techniques, such as FedCorr [5], leverage pair-wise cosine similarity or gradient projections to minimize redundancy and encourage diversity.

Why rethink client selection? A popular remedy is to select a subset of clients whose updates are “diverse”. Existing policies measure similarity—e.g., cosine distance or gradient norms—and then either pick the most similar (for faster convergence) or the most dissimilar (for exploration). Yet these second-order metrics ignore richer interactions that become pronounced under heterogeneous data: empirical studies show highly non-Gaussian gradients whose tails dominate optimization dynamics.

Our key idea. We introduce the symmetric co-skewness moment (SCKM), defined as

$$\mathrm{SCKM}\left(g_{i},g_{j}\right)=E\left[g_{i}^{2}g_{j}\right]+E\left[g_{i}g_{j}^{2}\right].$$

a third-order dissimilarity measure that rewards complementary gradients rather than merely similar ones. Counter-intuitively, favoring dissimilarity accelerates global convergence because it steers aggregation toward updates that correct each other’s biases. Clients reveal only a lightweight gradient sketch–a few random projections—before the server evaluates SCKM and decides who should train. To serve deployments of any size we provide two variants:

- SCKM-Select: ranks a small candidate pool directly by the SCKM score;
- SCKM-Cluster: applies a fast, elbow-guided clustering step, then runs SCKMSelect within clusters, scaling gracefully to thousands of users.

### Contributions

1. Moment-aware metric. We formulate SCKM as a symmetric co-skewness moment that captures third-order, tail-sensitive interactions among client gradients.
2. Communication-efficient selection. A gradient-sketch pipeline lets the server evaluate SCKM without full gradients, while an elbow rule adaptively balances diversity and bandwidth.
3. Algorithmic variants. We present SCKM-Select and the scalable SCKM-Cluster for small and large client pools, respectively.
4. Extensive empirical validation. On CIFAR-10, and Tiny-ImageNet with non-IID splits, both variants cut the rounds-to-target-accuracy by up to 30% and boost final accuracy by 1–3 percentage points over cosine-similarity, loss-sketch, and max-diversity baselines—without increasing communication.

These results highlight the power of moment-aware gradient dissimilarity for communication-efficient FL.

Notation: We use lowercase boldface letters (e.g., z) for vectors and calligraphic uppercase letters (e.g., A) for sets. For a set A, let |A| denote its cardinality. We adopt the shorthand [m:n]≜{m,…,n} and [n]≜{1,…,n}. Throughout, k∈[K] indexes clients, t∈[T] denotes communication rounds, $s\in S_{t}\subseteq \left[K\right]$ represents a selected client with $|S_{t}|=S$, and r∈[R] is a random seed.

## 2. Related Work

**Client Selection in Federated Learning.** Statistical heterogeneity is a fundamental challenge in federated learning (FL), arising from diverse and non-IID data distributions across clients. Devices such as smartphones, hospitals, and IoT sensors generate data under different operating conditions, user behaviors, and regional or task-specific contexts [6,7]. As a result, client datasets are often imbalanced, incomplete, or small, leading to biased updates, unstable convergence, and degraded generalization when naive aggregation strategies such as FedAvg are employed [8].

Client selection has emerged as a key mechanism to mitigate these effects by choosing a subset of informative participants in each training round, thereby improving both communication efficiency and optimization performance [9]. Early approaches primarily focused on system-level considerations, such as device availability, communication latency, and computational capacity. For instance, FedAvg implicitly relies on random sampling to balance load [8], while FedCS prioritizes clients based on their resource constraints to reduce straggler effects [10]. While effective from a systems perspective, these methods do not explicitly leverage statistical information about client data or updates.

More recent work incorporates statistical utility signals to guide client selection. Several methods prioritize clients with large local losses or update magnitudes, under the assumption that these clients contribute more significantly to global model improvement [3,4]. Other approaches use proxies such as data representativeness, gradient norms, or weight divergence to estimate client importance [2,11]. These criteria are typically easy to compute but rely on low-order statistics that may inadequately capture complex update distributions.

A complementary line of work explicitly compares clients using pair-wise similarity measures. FedSel [12] and FedCorr [5] exploit cosine similarity between local gradients to reduce redundancy among selected clients, encouraging diversity. GPFL [13] quantifies each client’s contribution via gradient projection outside the span of others, while GBME [14] introduces gradient proxies and applies cosine-based grouping. Relatedly, FedSim [15] detects divergent clients using gradient similarity and $l_{2}$ distances. Cluster-based FL methods [9] further organize clients into homogeneous groups to address non-IID data by enabling group-wise model updates.

Despite their effectiveness, most existing client-selection methods rely on geometric or second-order statistics such as norms, inner products, or cosine similarity. These metrics implicitly assume light-tailed or near-Gaussian gradient distributions and do not explicitly model higher-order statistical interactions among client updates.

**Heavy-Tailed Optimization Noise.** Recent empirical and theoretical studies indicate that deep neural network gradients are often non-Gaussian and heavy-tailed, particularly in non-IID and partial-participation regimes [16,17,18]. In FL, statistical heterogeneity across clients severely amplifies gradient variability, producing rare but extreme updates that can destabilize training and lead to abrupt performance degradation [19]. From a optimization theory standpoint, classical convergence analyses and low-order geometric metrics (such as variance boundaries or directional cosine similarities) misbehave under these power-law profiles because they fail to characterize volatile local curvature fluctuations and distributional asymmetry [20].

To safeguard convergence under such fat-tailed drift, a prominent line of contemporary research focuses on passive or localized mitigation. This paradigm has recently evolved from simple algebraic threshold clipping [19,21] to complete gradient normalization [20], mathematically proving that modifying or entirely discarding erratic local gradient magnitudes is a pre-requisite to secure bounded error trajectories. To establish tighter analytical guarantees without destroying gradient structures, recent optimization breakthroughs have introduced explicit high-order moment or curvature awareness—such as local Hessian variant clipping [22] and robust medoid mini-batch gradient sampling [23]—proving that regularizing higher-order geometric signals is mathematically required to shatter the notorious “heavy-tailed noise barrier” [24] that hampers standard stochastic solvers. Furthermore, recent mathematical frameworks have successfully extended these robust estimators to the gradient-free black-box domain, formulating stochastic zeroth-order optimization under heavy-tailed functional perturbations [25], while decentralized optimization models strive to regulate worst-case minimax-optimal drift limits [26].

**Our Approach.** Motivated by the above observations on heavy-tailed client updates and cross-client statistical heterogeneity, we propose a moment-aware client-selection framework that explicitly accounts for higher-order interactions among client updates. We introduce SCKM as a pair-wise similarity metric designed to emphasize higher-order cross-moments and improve sensitivity to rare but influential tail events under heavy-tailed regimes.

To maintain communication efficiency in heterogeneous federated settings, each participating client transmits a lightweight sketch consisting of sampled entries from a designated layer, rather than uploading full-dimensional model updates. Based on these compressed summaries, the server computes pair-wise SCKM similarities and selects clients to jointly promote statistical utility and update diversity in subsequent training rounds.

To support different deployment scales, we further consider two execution modes: (i) a direct ranking-based selection strategy for moderate client pools, and (ii) a scalable clustering-assisted variant that incorporates a lightweight *k*-medoids filtering stage to reduce redundancy among highly similar client updates. The mathematical formulation of SCKM and the detailed procedures of these selection strategies are presented in the following sections.

## 3. Problem Formulation

**Figure 1:**
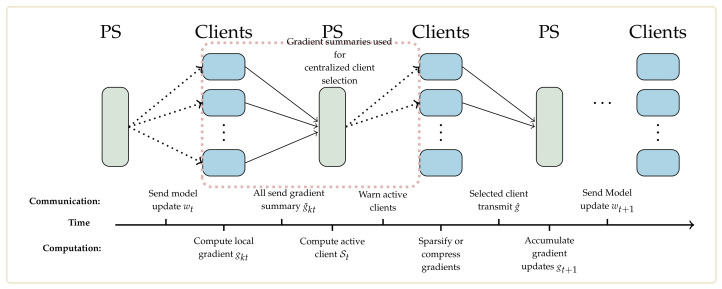
Gradient Summaries for Centralized Client Selection (GSCCS). At each round *t*, the parameter server (PS) broadcasts the current model $w_{t}$ to all available clients. Each client computes a local gradient $g_{kt}$ and transmits a lightweight summary $s_{kt}$ to the PS. Based on these summaries, the PS selects a subset of active clients $S_{t}$, which then transmit their full gradients. The PS aggregates the received updates to form ${\bar{g}}_{t}$ and updates the global model to $w_{t+1}$. The use of gradient summaries enables centralized, statistics-aware client selection with negligible communication overhead.

We consider an FL system in which a parameter server (PS) selectively activates a subset of clients at each communication round based on lightweight information about their local updates. We formalize this setting as Gradient Summaries for Centralized Client Selection (GSCCS), illustrated in Figure 1.

At round *t*, the protocol proceeds in two phases:

1. Summary transmission. Each client k∈[K] computes a compressed summary $s_{kt}=\phi \left(g_{kt}\right)$ of its local gradient $g_{kt}$ using a sketching function $\phi :R^{m}\to R^{p}$, and sends $s_{kt}$ to the PS.
2. Centralized selection. Based on the received summaries ${\left\{s_{kt}\right\}}_{k=1}^{K}$, the PS selects a subset $S_{t}\subseteq \left[K\right]$ of *S* clients for full participation.

Since p≪m, the summaries incur negligible communication overhead compared to transmitting full gradients. Once $S_{t}$ is selected, the PS aggregates the corresponding updates,

(1) $${\bar{g}}_{t}=\frac{1}{S}\sum_{k\in S_{t}}g_{kt},$$

and updates the global model via

(2) $$w_{t+1}=w_{t}-\eta_{t}{\bar{g}}_{t}.$$

We denote the client-selection rule by

(3) $$S_{t}=\pi \;\left({\left\{s_{kt}\right\}}_{k\in [K]}\right),$$

where π maps gradient summaries to a subset of active clients.

The objective of GSCCS is to minimize the performance degradation induced by partial participation. Formally, we seek sketching and selection rules (ϕ,π) that minimize the average loss gap relative to an oracle selection with full gradient access,

(4) $$\min_{\phi ,\pi }\;\frac{1}{T}\sum_{t=1}^{T}\left(L\left(w_{t}-\eta_{t}{\hat{g}}_{t}^{*}\right)-L\left(w_{t}-\eta_{t}{\hat{g}}_{t}\right)\right),$$

where ${\hat{g}}_{t}^{*}$ denotes the aggregate gradient of the optimal size-*S* client subset under full information.

This formulation highlights the central challenge addressed in this paper: designing summary-based selection policies that preserve informative gradient diversity under strict communication constraints.

### 3.1. Modeling Assumptions and Scope

The GSCCS formulation abstracts the client-selection problem by separating lightweight decision information from full gradient transmission. To keep the analysis and design tractable, we adopt the following standard and mild assumptions, which are consistent with common practice in FL literature and validated empirically in Section 6.

#### 3.1.1. Gradient Summaries

Each client transmits a compressed summary $s_{kt}=\phi \left(g_{kt}\right)$ of its local gradient, where the sketch dimension satisfies p≪m. The summary is assumed to preserve sufficient structural information to enable meaningful pair-wise comparison among clients. In practice, ϕ may correspond to random subsampling or projection, and we explicitly evaluate the impact of summary dimension in our experiments.

#### 3.1.2. Layer-Wise Treatment

Client selection and similarity computation are performed at the level of individual network layers. For analytical clarity, gradients from different layers are treated independently in the problem formulation, which can be interpreted as analyzing a single representative layer. In implementation, the proposed method is applied layer-wise to multi-layer networks, as detailed in Section 6.

#### 3.1.3. Per-Round User Selection

The optimal selection of clients over a finite training horizon is generally computationally intractable. Accordingly, we adopt a per-round greedy selection strategy based on the current gradient summaries. This choice reflects practical FL deployments in which users can freely join and leave the training process, so that long-term planning is generally not desirable.

#### 3.1.4. Stochastic Setting

Local gradients are inherently stochastic due to mini-batch sampling and random data distribution and network initialization. When discussing statistical properties of the gradient, we do not intend to provide a complete probabilistic model of the FL dynamics, but rather to define a principled and implementable setting in which summary-based client selection can be studied. The effectiveness of the proposed moment-aware criteria is demonstrated through extensive empirical evaluation under varying degrees of heterogeneity, participation budgets, and model architectures.

A summary of the notation introduced in this section is presented in Table 1.

## 4. Empirical Feature Ranking

To understand which pair-wise gradient metrics provide the most informative signal for client selection under heterogeneous data, we conduct a lightweight feature-ranking study. Rather than designing bespoke predictors, we use a simple logistic-regression probe to evaluate how well each candidate feature predicts the relative utility of client pairs across training rounds, heterogeneity levels, and random seeds. This evaluation serves solely as empirical guidance for metric design; the client-selection mechanisms proposed later do *not* require training any classifier.

We consider a family of pair-wise metrics capturing geometric similarity (e.g., cosine and $l_{p}$ distances) and higher-order statistics (e.g., marginal moments and co-moments). Definitions are summarized in Table 2. Table 3 reports the resulting average feature ranking (lower is better), while the corresponding relative-accuracy scores (Rel.A) are provided in Table 4. Across conditions, SCKM emerges as the most reliable predictor, motivating its use as the core dissimilarity measure in Section 5.

### 4.1. Logistic-Regression Probe for Pair-Wise Utility

We focus on the controlled setting of selecting *two* clients at a time. For each round *t* and each client pair $\left(k,k^{\prime }\right)$, we compute a feature value $f(g_{kt},g_{k^{\prime }t})$ (or a feature vector collecting multiple metrics), and associate it with a label measuring how beneficial that pair is. We then fit a logistic regressor to predict the label from the feature(s), and use its predictive loss as a standardized proxy for the quality of the feature.

Concretely, let $x_{k,k^{\prime }}$ denote the feature vector for pair $\left(k,k^{\prime }\right)$ and let $y_{k,k^{\prime }}\in \left(0,1\right)$ denote its normalized utility label. We estimate the logistic model parameters w by minimizing the binary cross-entropy

(5) $$L_{\mathrm{CS}}=\min_{w}\;\frac{1}{J}\sum_{\begin{array}{c} k,k^{\prime }\\ k\neq k^{\prime } \end{array}}\mathrm{BCE}\;\left(\sigma \left(w^{⊤}x_{k,k^{\prime }}\right),\;y_{k,k^{\prime }}\right),$$

where σ(·) is the sigmoid, $\mathrm{BCE}\left(\hat{y},y\right)=-y\log\hat{y}-\left(1-y\right)\log\left(1-\hat{y}\right)$, and *J* is the total number of observed pairs across all conditions.

#### Label Construction

For each experimental slice (round, heterogeneity level, and random seed), we evaluate all client pairs and record a scalar performance score (e.g., validation accuracy after updating with that pair). We then normalize these scores within the slice using min–max scaling to obtain labels $y_{k,k^{\prime }}\in \left(0,1\right)$, so that labels represent *relative* pair-wise utility under comparable conditions. The logistic-regression probe takes as input the pair-wise gradient features listed in Table 2. In particular, we instantiate the corresponding families using $d_{p}$ and $\cos_{p}$ with p∈{1,2,3,4}, and marginal moments $m_{k}$ with k∈{2,3,4} (along with the remaining geometric and statistical features defined in the same table). We repeat this procedure across rounds, shard configurations, and seeds, and pool the resulting labeled pairs to fit the probe.

We group candidate features into two classes:

- Deterministic (geometric) features: $d_{p}$, $s_{p}$, $d_{2,p}$, $\cos_{p}$, and ${\langle \cdot ,\cdot \rangle }_{p}$.
- Stochastic (moment-based) features: $C_{p}$ and $m_{k}$ (and SCKM, as a third-order cross-moment feature).

### 4.2. Ranking and Relative Accuracy

For each candidate feature *f*, we fit a logistic regressor using only that feature (or only the corresponding one-dimensional score) and record its average loss L(f) across all conditions. We define:

(6) $$\mathrm{Rank}\left(f\right)≜\mathrm{rank}\;\left(L(f)\right),$$

where smaller loss implies better rank (rank 1 is best). To report a normalized score comparable across settings, we also define the relative accuracy

(7) $$\mathrm{Rel}.A\left(f\right)≜\frac{L_{\max}-L\left(f\right)}{L_{\max}},\;L_{\max}≜\max_{f^{\prime }}L\left(f^{\prime }\right),$$

so that larger values indicate better predictive power.

### 4.3. Key Empirical Finding: SCKM Is Most Predictive

Table 3 shows that SCKM consistently exhibits competitive and frequently top-tier predictive performance across rounds and heterogeneity levels when compared with both geometric similarities (cosine, $l_{p}$ distances) and marginal moment-based features.

Cosine-based metrics perform adequately in mild heterogeneity, but their rank deteriorates as data become more imbalanced and skewed. By contrast, SCKM exhibits robust predictive performance across settings, supporting the use of higher-order cross-moments as a more informative signal for client selection under heterogeneous gradients.

Motivated by this evidence, we adopt SCKM as the core dissimilarity measure in the client-selection mechanisms developed in Section 5.

### 4.4. A Statistical Interpretation of SCKM

The empirical evidence in Section 4 suggests that SCKM captures informative structure in the interaction between client gradients, particularly under heterogeneous and heavy-tailed regimes. In this subsection, we provide a statistical interpretation that helps explain this behavior, without claiming optimality or completeness.

Our discussion is motivated by recent observations that gradient coordinates in deep networks are often heavy-tailed and poorly modeled by Gaussian laws [17,27]. Accordingly, we consider a stylized setting in which the marginal distribution of each gradient coordinate follows a generalized-Gaussian law, while dependencies across clients are introduced through a nonlinear coupling.

Specifically, we examine a simple two-client model in which latent independent generalized-Gaussian variables are coupled through a cubic interaction term—ρ. A full derivation of the CGG model and additional discussion are provided in Section 9.

**Figure 2:**
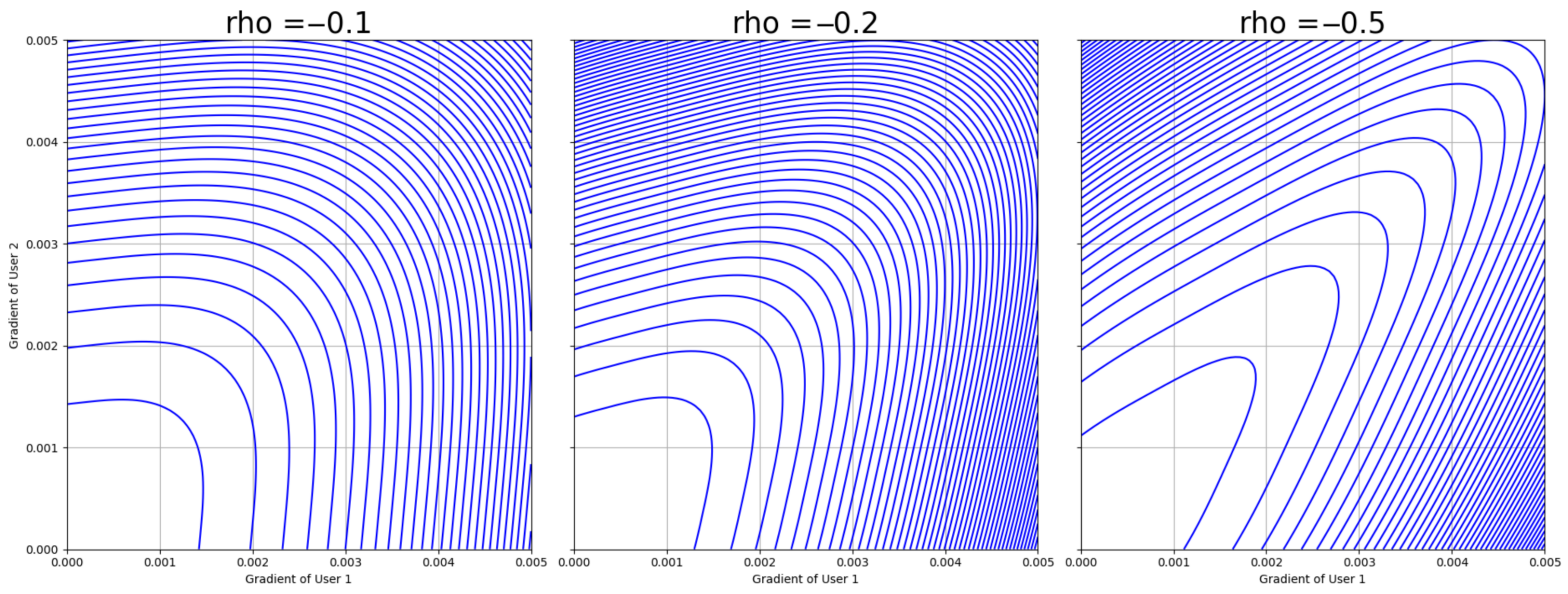
Illustration of joint heavy-tailed behavior induced by the coupling parameter ρ in a coupled generalized-Gaussian model (β=3) discussed in Section 4.4. Shown are contour lines of the joint density for increasing values of ρ. Larger coupling magnitudes produce sharper, elongated tails along specific directions, corresponding to rare but correlated large-gradient events. The figure is intended as an illustrative visualization of tail control rather than a quantitative distribution fit.

Figure 2 visualizes level sets of the joint density for different values of the coupling parameter ρ. As ρ increases in magnitude, the distribution departs from elliptical geometry and develops increasingly elongated, sharp tails along preferred directions. This effect reflects the emergence of rare but correlated large-gradient events, which are weakly visible in standard second-order statistics but become pronounced under higher-order interactions.

#### Interpretational Scope

We emphasize that the β-CGG construction is intended as an illustrative statistical interpretation rather than a proof of optimality of SCKM. Other higher-order statistics, including cumulant-based or normalized skewness measures, may also provide useful client-selection signals under heavy-tailed regimes.

## 5. Proposed Approach: SCKM-Based Client Selection

Guided by the empirical ranking in Section 4, we use SCKM as a dissimilarity measure between client updates. Intuitively, SCKM assigns a large score to client pairs whose gradient coordinates exhibit asymmetric co-fluctuations, which becomes informative in the heavy-tailed regimes often induced by non-IID data. We leverage this signal to schedule clients whose updates are complementary rather than merely similar.

We consider two deployment regimes: (i) SCKM-Select, a direct ranking rule suited to small/medium client pools, and (ii) SCKM-Cluster, a scalable variant that clusters clients using SCKM and then applies SCKM-Select within clusters.

### 5.1. SCKM Dissimilarity on Gradient Sketches

At each round *t*, client *k* computes a gradient sketch ${\tilde{g}}_{k}^{\left(t\right)}\in R^{d^{\prime }}$ with $d^{\prime }\;\ll \;d$ via random subsampling and transmits it to the server—as discussed in Section 3). Given two sketches $\tilde{g},{\tilde{g}}^{\prime }\in R^{d^{\prime }}$, the server computes

(8) $$\mathrm{SCKM}\left(\tilde{g},{\tilde{g}}^{\prime }\right)\;≜\;\frac{1}{d^{\prime }}\sum_{i=1}^{d^{\prime }}\left({\tilde{g}}_{i}{\left({\tilde{g}}_{i}^{\prime }\right)}^{2}+{\left({\tilde{g}}_{i}\right)}^{2}{\tilde{g}}_{i}^{\prime }\right),$$

where the coordinate-wise averaging provides a stable scalar statistic from compressed updates. (Other normalizations (e.g., standardizing by coordinate-wise scale estimates) are possible; we use Equation (8) for simplicity and robustness across architectures.) We treat SCKM as a dissimilarity score: larger values indicate more complementary gradient structure.

### 5.2. SCKM-Select: Direct Ranking for Small Client Pools

When the server can solicit sketches from all *K* clients, we compute pair-wise SCKM scores and rank clients by their average dissimilarity to the population. Specifically, define

(9) $$\eta_{k}^{\left(t\right)}\;=\;\frac{1}{K-1}\sum_{\begin{array}{c} k^{\prime }=1\\ k^{\prime }\neq k \end{array}}^{K}\mathrm{SCKM}\;\left({\tilde{g}}_{k}^{\left(t\right)},{\tilde{g}}_{k^{\prime }}^{\left(t\right)}\right),$$

and select the *B* clients with largest $\eta_{k}^{\left(t\right)}$. This rule favors clients whose updates are broadly complementary to others, encouraging gradient diversity while remaining extremely simple.

Algorithm 1 summarizes SCKM Select. If ties occur, they can be broken using an age-of-gradient (AoG) queue (e.g., prioritizing clients that have not participated recently).
**Algorithm 1** SCKM-Select Strategy**Input:** Client pool K (size *K*), Selection budget *B***Output:** Selected clients SServer collects gradient sketches ${\left\{{\tilde{g}}_{k}\right\}}_{k\in K}$**for** each client k∈K **do**   Calculate average dissimilarity $\eta_{k}$ via Equation (6)**end for**Sort clients by $\eta_{k}$ in descending orderS← Top-*B* clients with highest $\eta_{k}$**return** S

### 5.3. SCKM-Cluster: Scalable Selection for Massive Client Pools

For large *K*, computing all K2 pair-wise scores is prohibitive. We therefore introduce a two-stage scalable variant:

(a) Clustering (server side). Using the sketches ${\left\{{\tilde{g}}_{k}^{\left(t\right)}\right\}}_{k=1}^{K}$ and SCKM as a dissimilarity measure, the server partitions clients into ${\hat{C}}^{\left(t\right)}$ clusters via *k*-medoids. (*k*-medoids is preferable to *k*-means since  SCKMis not induced by a Euclidean geometry and medoids correspond to real clients.) The number of clusters ${\hat{C}}^{\left(t\right)}$ is determined by an elbow rule. At each communication round *t*, the server first determines a round-wise cluster cardinality ${\hat{C}}^{\left(t\right)}$ using an elbow criterion. The value ${\hat{C}}^{\left(t\right)}$ is therefore dynamic and may vary across training rounds. We define the temporally averaged operating cluster cardinality as
  (10) $$C^{*}=\left\lfloor \frac{1}{T}\sum_{t=1}^{T}{\hat{C}}^{\left(t\right)}\right\rfloor .$$
(b) Intra-cluster selection. Within each cluster *c*, the server applies SCKM-Select locally and chooses ⌈B|c|/K⌉ clients, ensuring a total of exactly *B* selected clients.

The resulting computation reduces from $O(K^{2})$ pair-wise scoring to

(11) $$O\left(Kd^{\prime }\right)\;+\;\sum_{c=1}^{{\hat{C}}^{\left(t\right)}}{O(|c|}^{2})\;\approx \;O\;\left(Kd^{\prime }+{\hat{C}}^{\left(t\right)}B^{2}\right),$$

which is linear in *K* up to small intra-cluster terms (since we only rank within clusters at selection-budget scale). Complexity Analysis. For SCKM-Select, the dominant computation arises from pair-wise SCKM evaluation among candidate clients. Given *K* clients and sketch dimension $d^{\prime }$, this requires $O(K^{2}d^{\prime })$ time. If all pair-wise scores are stored, the space complexity is $O(K^{2})$. In practice, however, the average dissimilarity score in Equation (9) can be accumulated online, reducing the auxiliary storage to O(K).

**Figure 3:**
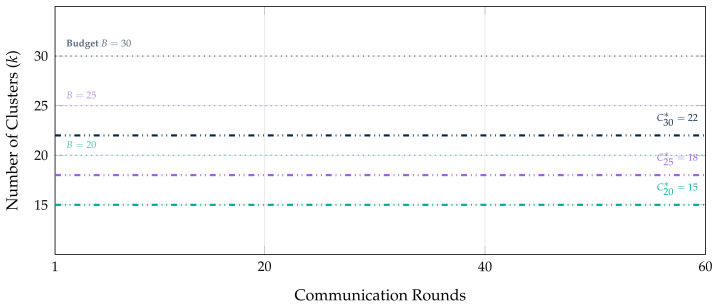
Dynamic evolution of the round-wise cluster cardinality ${\hat{C}}^{\left(t\right)}$ under varying sampling budgets *B* (S=1, K=50). The dotted horizontal lines represent the hard budget constraints *B*, while the dash-dotted lines indicate the budget-specific temporally averaged cluster cardinality $C_{B}^{*}$ defined in Equation (10) and determined via the elbow criterion.

For SCKM-Cluster, the server first partitions clients into ${\hat{C}}^{\left(t\right)}$ clusters and then performs SCKM-based selection within each cluster. The resulting time complexity is approximately

(12) $$O\left(Kd^{\prime }\right)+\sum_{c=1}^{{\hat{C}}^{\left(t\right)}}O(|C_{c}{|}^{2}d^{\prime }),$$

which scales more favorably than full pair-wise comparison when clusters are reasonably balanced. Compared with full-gradient-based or full-pair-wise client-selection strategies, the proposed framework operates only on compressed gradient sketches during the selection stage, yielding a communication cost of $O(Kd^{\prime })$ with $d^{\prime }\ll d$. This makes SCKM-Cluster particularly suitable for large client pools and resource-constrained federated deployments. Algorithm 2 lists the detailed steps. Specifically, we employ this mechanism to adaptively select the optimal cluster cardinality $C^{*}$ under varying sampling budgets *B*; a conceptual hint of this elbow-based selection logic is provided here, while the comprehensive numerical evaluation and stability analysis is visualized in Figure 3.
**Algorithm 2** SCKM-Cluster**Input:** Client pool K, Selection budget *B*, minimum cluster number $C_{\min}$**Output:** Selected clients $S_{total}$**Adaptive Cluster Determination:**Determine the round-wise cluster cardinality ${\hat{C}}^{\left(t\right)}$ via the elbow criterion over candidate values $C\in [C_{\min},B]$**Server-side Clustering:**Partition K into $\left\{C_{1},\ldots ,C_{{\hat{C}}^{\left(t\right)}}\right\}$ using *k*-medoids   *Metric: Pair-wise SCKM scores (Equation 8)$S_{total}\leftarrow \emptyset$**Intra-cluster Selection:****for** each cluster $c\in \{1,\ldots ,{\hat{C}}^{\left(t\right)}\}$ **do**   Determine local budget $b_{c}\leftarrow \left\lceil B\cdot |C_{c}|/K\right\rceil$   $S_{c}\leftarrow \mathrm{SCKM}-\mathrm{Select}\left(C_{c},b_{c}\right)$ Call Algorithm 1   $S_{total}\leftarrow S_{total}\cup S_{c}$**end for****return** $S_{total}$

**Remark** **1** (Select as a special case). *Setting ${\hat{C}}^{\left(t\right)}=1$ reduces SCKM-Cluster to SCKM-Select.*

## 6. Numerical Evaluations

### 6.1. Experimental Setup

Datasets and Models. We conduct experiments on both CIFAR-10 and CIFAR-100 classification benchmarks. For CIFAR-10, we use a VGG16 backbone adapted to the 10-class setting by modifying the final classifier layers. For CIFAR-100, we similarly adapt VGG16 to 100 classes.

Gradient Summaries. In all simulations, the size of the gradient summary is chosen as 0.5% of the gradient size. The elements in the gradient summary are randomly selected at each iteration.

Data Partitioning (Shards). To emulate statistical heterogeneity across clients, we adopt a non-IID partitioning strategy inspired by [1]. The training data is first sorted by label and then divided into equal-sized segments, referred to as shards. Each client is assigned *S* shards at random. Smaller values of *S* correspond to higher heterogeneity: e.g., S=1 means a client holds data from a single class, whereas S=5 provides a more balanced distribution.

Protocol and Baselines. Unless otherwise stated, all clients participate in local training for a fixed number of mini-batches per round. We compare against leading selection heuristics that use geometric similarity, gradient projection, or clustering (e.g., GPR, AFL, etc.). We adopt the same training hyperparameters across methods for fair comparison.

Parameter Selection. The shard configurations and client-selection budgets are chosen to emulate practical federated learning regimes with varying degrees of statistical heterogeneity and communication constraints. Smaller shard counts correspond to more severe non-IID client distributions, while larger selection budgets improve participation diversity at the expense of increased communication overhead. The gradient-summary ratio is fixed to 0.5% to maintain lightweight communication while preserving sufficient structural information for pair-wise client comparison. These settings allow us to systematically evaluate the robustness of SCKM under different levels of heterogeneity and participation sparsity.

### 6.2. SCKM-Select on CIFAR-10

**Figure 4:**
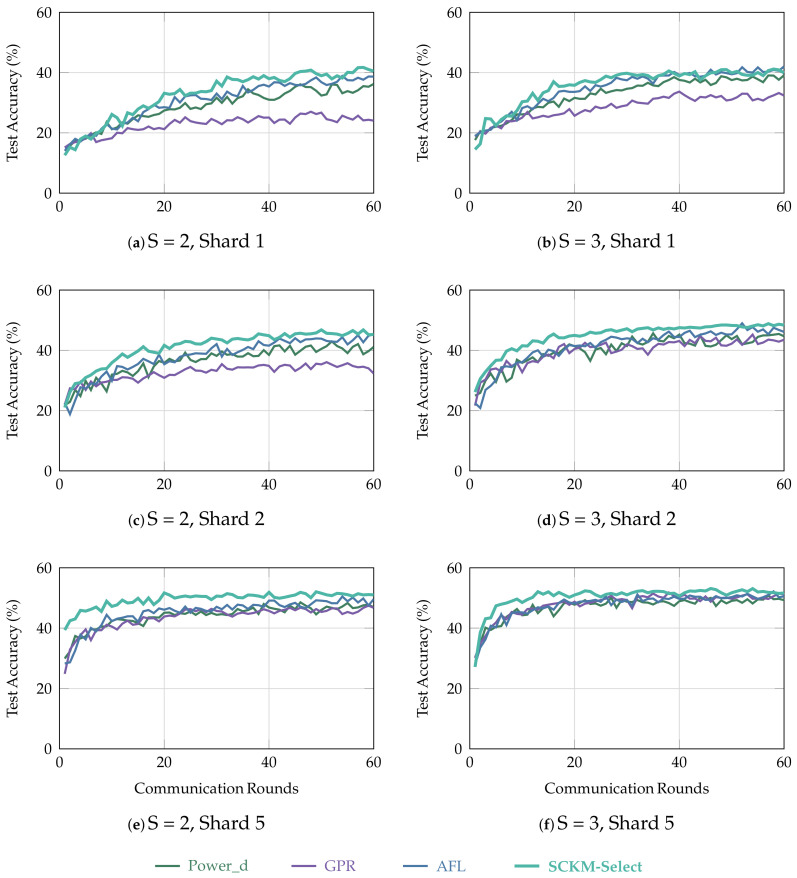
Client selection performance comparison using subfloat format.

Figure 4 summarizes the performance of SCKM-Select on CIFAR-10 with 10 clients under varying degrees of statistical heterogeneity (S∈{1,2,5}). Each subplot corresponds to a specific shard configuration and client-selection budget (e.g., selecting 2 or 3 clients per communication round).

Several consistent quantitative trends can be observed across all settings. First, the performance advantage of SCKM becomes more pronounced under strongly heterogeneous regimes with smaller shard counts (S=1 or S=2), where conventional low-order geometric similarity measures exhibit reduced stability and slower convergence. Second, increasing the number of selected clients generally improves convergence stability and final test accuracy by increasing participation diversity during aggregation. Across nearly all configurations, SCKM-Select achieves faster convergence and higher final accuracy than the compared baselines while maintaining smoother optimization trajectories throughout training. These observations suggest that higher-order moment-aware client selection is particularly beneficial under heterogeneous federated optimization settings where asymmetric cross-client gradient interactions become significant.

To further quantify the comparative improvements of SCKM-Select, Table 5 reports average test accuracies for Select = 2 and Select = 3 across different shard configurations and training iterations. Across most settings, SCKM-Select achieves the highest average accuracy among the compared methods while maintaining competitive variance statistics, indicating both effectiveness and stable optimization behavior relative to AFL, FedCor, and Power_d baselines.

### 6.3. SCKM-Cluster on CIFAR-100

To evaluate scalability under stronger heterogeneity and larger client populations, we further extend experiments to CIFAR-100 with 50 users, where each client holds only 1–2 shards, resulting in highly non-IID local distributions. Compared with CIFAR-10, this setting introduces substantially higher gradient diversity and optimization instability. To improve scalability and reduce redundancy among highly similar client updates, we therefore employ SCKM-Cluster, which first groups clients via elbow-guided clustering under the SCKM dissimilarity metric and then performs representative client selection within each cluster.

Figure 3 illustrates the evolution of the adaptive cluster cardinality under different communication budgets. The results show that the round-wise cluster number ${\hat{C}}^{\left(t\right)}$ remains relatively stable throughout training despite temporary fluctuations caused by heterogeneous client updates and stochastic gradient noise.

Several consistent trends can be observed. First, larger communication budgets generally lead to larger operating cluster cardinalities, reflecting increased participation diversity during aggregation. Second, the elbow-guided clustering mechanism consistently converges to effective cluster structures below the maximum budget limit, indicating that many client updates remain statistically redundant under highly non-IID settings. For example, under the extreme heterogeneity configuration (S=1, K=50), the method stabilizes around $C_{30}^{*}=22$ even when the communication budget allows selecting up to 30 clients.

These observations suggest that SCKM-Cluster effectively balances diversity preservation and redundancy reduction under heterogeneous federated optimization, while maintaining stable behavior across communication rounds.

Table 6 provides a quantitative comparison of SCKM-Cluster against several baselines (Kmeans_L2, AFL, gpr, and Power_d) across different shard configurations (*S*) and client-selection budgets (B=2,4,6). For each selection budget, we report test accuracy across multiple communication rounds together with the overall average and standard deviation.

Across most configurations, SCKM-Cluster achieves competitive or highest average accuracy among the compared methods, particularly under strongly heterogeneous settings (S=1 and S=2). The advantage becomes more visible as the communication budget increases, indicating that the clustering-assisted strategy benefits from increased participation diversity while still filtering redundant client updates. In addition to improved average accuracy, the reported variance statistics indicate that SCKM-Cluster maintains stable optimization behavior across training iterations under highly non-IID federated conditions.

**Figure 5:**
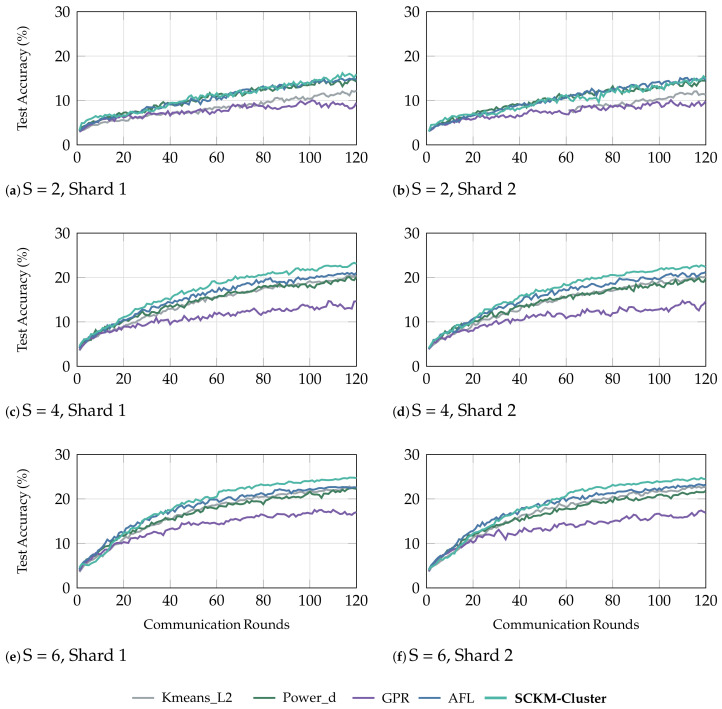
Client-selection performance across different shard levels (rows) and selection sizes (columns). Our method, SCKM-Cluster, consistently improves accuracy over baselines.

These numerical observations complement the convergence trends shown in Figure 5, suggesting that elbow-guided clustering under the proposed SCKM dissimilarity improves client-selection quality and scalability in heterogeneous federated learning scenarios.

Overall, the experiments demonstrate that higher-order moment-aware dissimilarity provides an effective and scalable criterion for client selection under diverse federated optimization regimes.

## 7. Conclusions

In this paper, we introduced the symmetric co-skewness moment (SCKM)—a third-order, moment-aware dissimilarity metric for client selection in federated learning under heterogeneous data distributions. Unlike traditional geometric or norm-based selection criteria, SCKM explicitly captures asymmetric co-moment structure in gradient updates, enabling the scheduler to identify clients whose contributions are complementary rather than redundant.

We proposed two practical algorithms built on this insight: SCKM-Select, which directly ranks clients based on pair-wise dissimilarity, and SCKM-Cluster, which introduces lightweight, elbow-guided clustering for scalability to large user populations. Across extensive experiments on CIFAR-10 and CIFAR-100 benchmarks, our methods consistently outperformed leading baselines in both convergence speed and final test accuracy—particularly under severe non-IID conditions where classical similarity measures tend to fail.

The numerical evaluations and aggregated summaries demonstrate that SCKM not only improves selection quality but also yields stable and robust performance across shard configurations and selection budgets. By tying moment statistics to practical selection mechanisms, this work bridges the gap between statistical gradient characterization and efficient client scheduling in federated systems.

Despite the encouraging empirical results, the current evaluation remains limited to controlled federated simulations using VGG-based image-classification benchmarks. In addition, the proposed framework relies on compressed gradient sketches, which may discard part of the fine-grained gradient structure during client comparison. Although the clustering-assisted variant improves scalability, higher-order moment estimation still introduces additional pair-wise computation compared with lightweight first-order similarity metrics. Nevertheless, the proposed framework is designed to operate with lightweight gradient summaries and limited communication budgets, making it naturally compatible with resource-constrained federated environments.

For future work, we plan to extend the theoretical analysis of SCKM under broader gradient distribution models, explore adaptive sketching strategies that further reduce communication overhead, and investigate the applicability of moment-aware client selection in transformer-based federated workloads, particularly under low-rank adaptation settings such as LoRA where structured gradient interactions may become especially informative. Evaluating SCKM under real-device deployment and practical edge-terminal conditions also remains an important direction for future research.

## 8. Relative Accuracy

In Section 4, we evaluated various pair-wise features using a logistic-regression probe to estimate their predictive power as client-selection signals. Alongside ranking metrics, another way to quantify feature quality is via relative accuracy, which measures how much each feature reduces the logistic loss relative to the worst-performing signal. Higher values indicate stronger utility for discriminating between informative and uninformative client pairs.

Formally, given a feature-based loss L(f) and the worst loss among all candidates, we define:

(13) $$\mathrm{Rel}.A\left(f\right)\;≜\;\frac{L_{\max}-L\left(f\right)}{L_{\max}},$$

where $L_{\max}=\max_{f^{\prime }}L\left(f^{\prime }\right)$ over the complete feature set.

Table 4 reports relative accuracy for each candidate feature across two perspectives: (i) across different data shard configurations, and (ii) across iterations during training. In both dimensions, the relative accuracy provides a normalized measure of how effectively the feature predicts client-pair utility relative to the baseline.

Across shard configurations, SCKM achieves the highest relative accuracy for S=1 and S=2, indicating its superior ability to reduce loss in the most heterogeneous settings. For higher shard counts (S=5), several moment-based features remain competitive, yet SCKM’s performance remains on par with or above the geometric measures.

When viewed over iterations, we observe that certain moments (e.g., $m_{1},m_{2},m_{3},d_{2}^{2}$) can occasionally outperform simple cosine variants at particular training stages, suggesting that moment structure provides useful information beyond directional similarity. However, SCKM remains competitive across iteration levels while providing consistently strong performance under the most heterogeneous shard configurations, reinforcing its utility as a robust higher-order feature.

At the aggregate level (“All”), the average relative-accuracy statistics indicate that higher-order moment-based features generally outperform purely geometric similarity measures across heterogeneous settings. In particular, SCKM demonstrates competitive overall predictive quality while remaining especially effective under strongly non-IID shard configurations. These relative-accuracy results complement the ranking analysis in Section 4, providing an alternative normalized perspective on feature utility for client-selection prediction.

## 9. Coupled Generalized-Gaussian Construction

To further motivate the choice of SCKM as a moment-aware dissimilarity measure, it is helpful to consider a flexible family of joint models that extends beyond simple Gaussian assumptions. In particular, we introduce a bivariate generalized-Gaussian construction that captures heavy tails and asymmetric co-dependence between gradients—properties often observed in deep learning updates under heterogeneous data.

Let $G_{1}$ and $G_{2}$ denote the (scalar) gradient coordinate values for two clients, each marginally following a zero-mean generalized normal distribution with scale parameters $\sigma_{1},\sigma_{2}>0$ and shape parameter β=3. A simple coupling between these marginals can be defined via the transformation

(14) $$\begin{array}{cc} X_{1} & =\sigma_{1}\left(\frac{G_{1}}{\sigma_{1}}+\rho \;\frac{G_{2}}{\sigma_{2}}\right), \end{array}$$

(15) $$\begin{array}{cc} X_{2} & =\sigma_{2}\left(\frac{G_{2}}{\sigma_{2}}+\rho \;\frac{G_{1}}{\sigma_{1}}\right), \end{array}$$

where ρ∈[−1,1] controls the strength of coupling. Under this construction, one obtains the following joint density for $\left(G_{1},G_{2}\right)$:

(16) $$\begin{array}{cc} & P_{G_{1},G_{2}}\left(g_{1},g_{2}\right)=\frac{4}{3^{2/3}\pi }\;\Gamma \;\left(\frac{2}{3}\right)\;\\ & \exp\;\left(-\left[{\left|\frac{g_{1}+\rho \;g_{2}\;\sigma_{1}/\sigma_{2}}{\sigma_{1}}\right|}^{3}+{\left|\frac{g_{2}+\rho \;g_{1}\;\sigma_{2}/\sigma_{1}}{\sigma_{2}}\right|}^{3}\right]\right), \end{array}$$

where Γ(·) denotes the gamma function. This expression can be expanded to show explicit dependence on the skewing parameter ρ:

(17) $$\begin{array}{c} P_{G_{1},G_{2}}\left(g_{1},g_{2}\right)=\frac{4}{3^{2/3}\pi }\;\Gamma \;\left(\frac{2}{3}\right)\;\\ \;\;\exp\;(-[\frac{\left(1+\rho^{3}\right)\left(\sigma_{2}^{3}\;g_{1}^{3}+\sigma_{1}^{3}\;g_{2}^{3}\right)}{\sigma_{1}^{3}\;\sigma_{2}^{3}}\\ \;\;\;\;\;\;\;\;\;\;\;\;\;\;\;\;\;\;\;\;\;+3\;\rho \;\frac{g_{1}^{2}g_{2}\;\sigma_{2}+g_{1}g_{2}^{2}\;\sigma_{1}}{\sigma_{1}\;\sigma_{2}}]), \end{array}$$

which introduces explicit third-order interaction terms between coordinates. When ρ=0, the two coordinates are independent generalized normals, and the joint reduces to the product of marginals. Nonzero ρ introduces asymmetric co-dependence that is not captured by second-order statistics alone.

We refer to this family as the β-Coupled Generalized-Gaussian (β-CGG) distribution with coupling parameter ρ and shape β=3. This model provides a simple yet expressive way to capture heavy tails and co-skewed dependencies between client gradient coordinates.

Under the β-CGG model, measures that incorporate third-order joint moments—such as SCKM—can be interpreted as statistically meaningful descriptors of asymmetric structure in the joint distribution. For example, the term $E[G_{1}^{2}G_{2}]$ (and its symmetric counterpart) appear directly in the exponent, suggesting their relevance for capturing gradient interactions beyond norms or directional cosine metrics. Although rigorous theoretical analysis of optimal dissimilarity measures under this model is beyond the scope of the present work, the β-CGG construction provides a statistical lens through which the empirical effectiveness of SCKM can be interpreted. A systematic characterization of theoretically optimal higher-order client-selection statistics remains an important direction for future work.

In higher dimensions, one may extend the β-CGG coupling by introducing a symmetric coupling matrix P that encodes pair-wise interactions across clients. A systematic study of such couplings and their relationship to selection heuristics is an interesting avenue for future research.

## Figures and Tables

**Table 1:** Summary of notation introduced in Section 3.

Notation	Quantity
*k*/*K*	Client index/Total number of clients
*t*/*T*	Round index/Total number of rounds
$w_{t}$	Global model parameters at round *t*
$\eta_{t}$	Learning rate at round *t*
$g_{kt}$	Local gradient of client *k* at round *t*
$s_{kt}$	Gradient summary of client *k* at round *t*
ϕ(·)	Sketching function
*m*	Gradient dimension
*p*	Summary (sketch) dimension
$S_{t}$	Selected client set at round *t*
*S*	Number of selected clients per round
π(·)	Client-selection policy
${\bar{g}}_{t}$	Aggregated gradient at round *t*
${\hat{g}}_{t}^{*}$	Oracle aggregated gradient (full information)

**Table 2:** Definitions of pair-wise gradient features used in Table 3.

Feature	Formula
$l_{p}$ Distance	$d_{p}\left(g_{i},g_{j}\right)={∥g_{i}-g_{j}∥}_{p}$
*p*-Norm Cosine	$\cos_{p}\left(g_{i},g_{j}\right)=\frac{{\langle g_{i},g_{j}\rangle }_{p}}{∥g_{i}{∥}_{p}{∥g_{j}∥}_{p}}$
*p*-Inner Product	${\langle u,v\rangle }_{p}=\frac{{∥u+v∥}_{p}-{∥u-v∥}_{p}}{4}$
Marginal *k*-Moments	$m_{k}\left(g_{i},g_{j}\right)=\frac{1}{d}\sum_{l=1}^{d}\left(g_{i,l}^{\;k}+g_{j,l}^{\;k}\right)$
SCKM	$\mathrm{SCKM}\left(g_{i},g_{j}\right)=\frac{1}{d}\sum_{l}\left(g_{i,l}^{2}g_{j,l}+g_{i,l}g_{j,l}^{2}\right)$

**Table 3:** Empirical feature ranking (higher is better) for pair-wise client-selection predictors, averaged across training rounds, heterogeneity levels, and random seeds.

Charac.	Type	$\cos_{1}$	$\cos_{2}$	$\cos_{3}$	$\cos_{4}$	$d_{2}$	$d_{2}^{2}$	$m_{1}$	$m_{2}$	$m_{3}$	$m_{4}$	SCKM
Shard	1	8.25	7.50	5.50	5.55	4.80	1.70	6.85	2.70	1.65	2.20	8.30
	2	8.45	7.90	6.30	5.35	4.05	1.60	5.00	2.90	2.95	2.55	7.95
	5	7.45	5.70	6.05	4.05	4.35	1.85	7.05	3.70	7.75	2.20	4.85
Iter.	1	7.33	8.33	6.00	5.00	4.33	0.33	7.00	4.33	2.33	2.00	8.00
	3	7.33	7.67	7.00	3.67	4.67	2.00	5.67	3.33	3.67	2.33	7.67
	5	8.00	5.33	6.33	3.67	7.00	2.00	7.33	1.33	4.67	1.33	8.00
	10	3.33	2.00	3.33	1.33	1.80	1.80	1.80	1.80	1.80	1.80	1.80
	15	4.00	2.00	2.33	1.67	2.00	2.00	2.00	2.00	2.00	2.00	2.00
All	Avg	8.05	7.03	5.95	4.98	4.40	1.72	6.30	3.10	4.12	2.32	7.03
	Std	1.28	1.48	1.19	1.23	1.25	0.98	1.26	1.55	1.54	1.32	1.14

**Table 4:** Relative accuracy (Rel.A) of individual pair-wise features, defined as the normalized reduction in logistic-regression loss with respect to the worst-performing feature. Bold entries indicate the highest relative accuracy for a given row.

Charac.	Type	$\cos_{1}$	$\cos_{2}$	$\cos_{3}$	$\cos_{4}$	$d_{2}$	$d_{2}^{2}$	$m_{1}$	$m_{2}$	$m_{3}$	$m_{4}$	SCKM
Shard	1	0.4326	0.4276	0.4268	0.4230	0.4779	0.3649	0.5110	0.3592	0.3699	0.4056	0.5789
	2	0.1765	0.1811	0.1790	0.1753	0.3483	0.3540	0.3044	0.3110	0.3021	0.3065	0.4541
	5	0.1042	0.0963	0.1091	0.1148	0.2255	0.2855	0.3430	0.3673	0.3715	0.3569	0.3030
Iter.	1	0.1048	0.1115	0.1218	0.1260	0.2015	0.2235	0.5007	0.4642	0.4245	0.4591	0.2938
	3	0.0970	0.1259	0.1359	0.1452	0.1747	0.3030	0.4542	0.4014	0.3968	0.3579	0.2858
	5	0.0739	0.0892	0.1294	0.1205	0.1698	0.3726	0.3384	0.4162	0.3753	0.3333	0.2376
	10	0.1189	0.1047	0.1209	0.1028	0.2218	0.3077	0.2958	0.3944	0.3954	0.3930	0.2313
	15	0.0829	0.0524	0.0410	0.0389	0.2414	0.2602	0.2744	0.3260	0.3459	0.3267	0.3632
All	Avg	0.1042	0.0963	0.1091	0.1148	0.2255	0.2855	0.3430	0.3673	0.3715	0.3569	0.3030
	Std	0.0258	0.0239	0.0367	0.0400	0.0671	0.0409	0.0951	0.0640	0.0784	0.0762	0.0754

**Table 5:** Comparison of S=2 vs. S=3 using SCKM-Select, AFL, FedCor, and Power_d under various experimental settings.

Charac.	Type	Select = 2				Select = 3			
		SCKM	AFL	FedCor	PD	SCKM	AFL	FedCor	PD
Shard	1	32.83	30.70	22.77	28.80	35.80	34.94	28.57	32.83
	2	41.21	38.15	32.62	36.36	44.85	41.57	39.86	40.14
	5	49.55	45.34	43.55	44.11	50.45	47.75	47.98	46.99
Iteration	15	39.19	33.80	30.54	33.50	43.49	39.30	37.21	38.44
	30	43.84	40.72	34.24	38.07	45.99	43.59	40.16	41.60
	45	45.40	42.77	34.72	40.05	46.35	44.63	41.81	42.85
	60	45.59	44.54	34.36	41.44	46.64	46.24	42.36	44.36
All	Avg	41.20	38.06	32.65	36.42	45.12	43.44	40.39	41.31
	Std	6.89	7.49	8.49	6.51	1.35	2.89	2.16	2.45

**Table 6:** Comparison of Shard 1 vs. Shard 2 using SCKM-Cluster, Kmeans_L2, AFL, gpr, and Power_d across Select values = 2, 4, 6. Bold values indicate best performer in each group.

Charac.	Type	Select = 2					Select = 4					Select = 6				
		SCKM	KM	AFL	gpr	PD	SCKM	KM	AFL	gpr	PD	SCKM	KM	AFL	gpr	PD
Shard	1	10.79	8.22	10.49	7.62	10.45	17.10	14.45	15.73	11.00	14.71	18.81	16.87	17.95	13.78	16.72
	2	10.11	9.81	10.39	7.46	10.24	16.91	14.47	15.84	11.03	14.58	18.63	16.94	18.11	13.30	16.53
Iteration	10	6.29	4.89	5.35	5.41	5.52	8.00	7.22	7.94	6.99	7.62	7.12	7.46	8.70	7.81	8.47
	20	6.84	5.58	6.60	5.98	7.18	10.61	9.29	10.61	8.49	10.17	12.27	11.16	12.54	10.23	11.68
	30	7.35	6.47	8.13	6.83	8.20	13.94	11.25	12.68	9.84	11.33	15.44	13.55	15.63	12.29	14.00
	40	8.75	6.93	9.11	6.51	9.10	15.43	12.75	14.38	9.89	13.70	17.46	15.71	17.28	13.09	15.18
	60	11.44	8.46	10.39	7.35	10.83	18.51	15.41	16.96	11.26	15.53	20.59	18.45	19.69	14.25	17.82
	80	12.47	8.91	12.93	8.60	12.83	20.61	17.33	18.81	12.07	17.87	23.15	20.49	21.23	15.67	19.05
	100	13.26	10.37	14.02	9.56	13.31	21.80	19.13	19.82	13.03	18.34	23.92	21.81	22.32	16.69	21.30
	120	15.42	11.74	14.61	9.68	14.35	22.72	20.34	21.12	14.64	19.92	24.61	22.59	22.89	16.95	22.02
All	Avg	10.23	7.92	10.14	7.49	10.17	15.29	14.09	15.29	10.78	14.31	17.54	16.40	17.54	13.37	16.19
	Std	3.38	2.38	3.45	1.62	3.17	4.68	4.73	4.68	2.48	4.36	5.02	5.40	5.02	3.20	4.73

## Data Availability

The data used in this study are publicly available. CIFAR-10 and CIFAR-100 can be accessed from their official repositories.
